# Editors’ role in shaping the publishing environment and guiding authors in the era of artificial intelligence

**DOI:** 10.3325/cmj.2024.65.471

**Published:** 2024-12

**Authors:** Hrvoje Barić, Lea Škorić, Svjetlana Kalanj Bognar

**Affiliations:** 1 *Croatian Medical Journal*, Zagreb, Croatia *hrvoje.baric@cmj.hr*; 2 Central Medical Library, Zagreb University School of Medicine, Zagreb, Croatia

A mere two years have passed since the first generative pre-trained transformer (GPT) was made available to the general public (1). Artificial intelligence (AI)-assisted technology has become the most transformative technology across the spectrum of human activity, academic publishing included. Almost immediately after the launch of GPT, the medical publishing community reacted by developing guidelines for authors on the use of AI in scientific writing and producing research articles. The *Croatian Medical Journal* first published on the topic in its February 2023 issue, with an editorial on opportunities and risks of AI in academic publishing (2). The June 2023 issue featured an AI-generated cover image that illustrated the editorial on the ever-changing nature of medicine and society (3). Although this was “only” a year ago, AI has profoundly changed the ways we design, conduct, report, and publish science. Some of these topics were tackled by a paper published in our June 2023 issue, which assessed the reliability of AI detection tools in scientific writing (4).

## *Status quo* among general medical journals

To find out to what extent medical journals have endorsed guidelines for authors on the use of AI in scientific writing, we screened the Clarivate Journal Citation Reports to identify all journals within the category of “Medicine, general and internal” indexed in the Science Citation Index Expanded, which also includes the *Croatian Medical Journal*. We identified 168 journals and checked whether they offered guidance for authors on AI usage. The majority (n = 135, 80.4%) of journals either provided explicit guidelines on AI usage or endorsed guidelines published by professional bodies such as the World Association of Medical Editors (WAME), International Committee of Medical Journal Editors (ICMJE), or Committee on Publication Ethics (5-7). There seems to be a higher awareness of and guidance on AI tools for authors among higher-ranking journals. Namely, a back-of-the-envelope calculation shows that 72 (85%) Q1, 37 (79%) Q2, and 21 (70%) Q3 journals offer guidelines for authors on AI (this should be taken with a grain of salt owing to the latest changes in quartile rankings, which distort symmetry). Overall, it seems that medical journal editors were quick to adapt to the new reality and adopt the role of policy developers.

## The *Croatian Medical Journal* endorses ICMJE AI guidelines

The increasing permeation of AI into the publishing environment calls for action. We as editors can adopt a clear position and offer guidance for authors. Therefore, we at the *Croatian Medical Journal* are amending our Guidelines for Authors, to include a section on the use of AI, in which we endorse the ICMJE guidelines (6). Some points to be emphasized from the ICMJE document pertain to: i) general remarks – the authors should disclose and detail AI use in manuscript production in both the letter to the editor and the manuscript; ii) authorship – AI does not meet authorship criteria as it cannot assume author responsibilities, however its use should be reported in the acknowledgment section; iii) the use of AI – human authors are responsible for all submitted content, including the AI-generated one; iv) journal editors and reviewers – using AI to review manuscripts might violate confidentiality, and both editors and reviewers should be aware of the fact.

To emphasize the importance of the guidelines the *Croatian Medical Journal* is endorsing, we have explored a hypothetical scenario. If the guidelines on the AI use are ignored and AI is used without disclosure in all stages of article production, from the study conception to the last edit, then the entire process can be performed without any human intervention, apart from occasional mouse clicks. At the same time, the humans overseeing the process are unaware of the underlying mechanics, biases, echo chambering, and tunnel visioning. All this, ultimately, to the detriment of researchers, authors, editors, and patients.

It took mere weeks for GPT to set the record for the fastest adoption of technology in history (8). The academic publishing community reacted swiftly, as is evident from the almost immediate development of guidelines on the use of this technology and, as we have shown by a short survey, a wide implementation of these guidelines by publishers and journals. The *Croatian Medical Journal* keeps up with the times by joining the community of medical journals that guide their contributors on the use of AI tools.
